# Modelling Soluble Solids Content Accumulation in ‘Braeburn’ Apples

**DOI:** 10.3390/plants10020302

**Published:** 2021-02-05

**Authors:** Konni Biegert, Daniel Stöckeler, Roy J. McCormick, Peter Braun

**Affiliations:** 1 Kompetenzzentrum Obstbau Bodensee, Fachgebiet Ertragsphysiologie, 88213 Ravensburg, Germany; mccormick@kob-bavendorf.de; 2 TUM School of Life Sciences, Technische Universität München, 85354 Freising, Germany; d.stoeckeler@posteo.de; 3 Institut für Obstbau, Hochschule Geisenheim University, 65366 Geisenheim, Germany; peter.braun@hs-gm.de

**Keywords:** Vis/NIR, repeated longitudinal measurements, apple maturation, precision horticulture

## Abstract

Optical sensor data can be used to determine changes in anthocyanins, chlorophyll and soluble solids content (SSC) in apple production. In this study, visible and near-infrared spectra (729 to 975 nm) were transformed to SSC values by advanced multivariate calibration models i.e., partial least square regression (PLSR) in order to test the substitution of destructive chemical analyses through non-destructive optical measurements. Spectral field scans were carried out from 2016 to 2018 on marked ‘Braeburn’ apples in Southwest Germany. The study combines an in-depth statistical analyses of longitudinal SSC values with horticultural knowledge to set guidelines for further applied use of SSC predictions in the orchard to gain insights into apple carbohydrate physiology. The PLSR models were investigated with respect to sample size, seasonal variation, laboratory errors and the explanatory power of PLSR models when applied to independent samples. As a result of Monte Carlo simulations, PLSR modelled SSC only depended to a minor extent on the absolute number and accuracy of the wet chemistry laboratory calibration measurements. The comparison between non-destructive SSC determinations in the orchard with standard destructive lab testing at harvest on an independent sample showed mean differences of 0.5% SSC over all study years. SSC modelling with longitudinal linear mixed-effect models linked high crop loads to lower SSC values at harvest and higher SSC values for fruit from the top part of a tree.

## 1. Introduction

In apple fruit production, tree physiological status, the farmer’s management decisions in the orchard, together with environmental factors influence postharvest fruit quality and storage pack-out. More specifically, factors that affect fruit quality can include crop load [1,2], timing of harvest [3], application of calcium and potassium fertilizer [4,5], light distribution within the orchard and temperature during important growth periods [6,7] as well as single tree or tree sector physiology [3].

Many factors within apple fruit tissues (cells per apple, energy status, cell wall stability [8]) which can determine harvest date and storage pack-out cannot be seen from the outside of the fruit. Depending on the wavelength, optical sensors (visible (Vis) and near-infrared (NIR) point spectroscopy) can help to get a non-destructive view of the fruit from 1–2 cm under the skin [9]. These portable optical sensors are now relatively inexpensive and fast [10]. In addition, data handling and chemometric software are user friendly (own experience). Light reflectance from the fruit can be monitored in the field to give information about plant pigment development such as chlorophyll, anthocyanins and carotinoids in the Vis spectra [11]. In addition, partial least squares regression (PLSR) modelling for the NIR spectra can be used to estimate soluble solids content (SSC) and dry matter content [12,13,14]. Further information on fruit tissues can be obtained from the light scattering of cell walls and other cellular components using more advanced technologies like spatial frequency domain imaging [15,16]. However, the latter laboratory based technology is not available for applied field-sensing to separate the absorbance from the scattering coefficient. One of the advantages of non-destructive sensor technology is the possibility to gain a large data set on a small-orchard scale during fruit maturation and link these spectral data together with other temporal and orchard field data. Biological and spatial variation are typically high, even within a small-scale apple orchard [17,18]. Moreover, fruit should be harvested and managed according to orchard variation such as site and cropping history to maintain the best possible fruit quality after long-term controlled atmosphere storage [19].

The development of SSC in individual apples depends mainly on the light distribution within the planting system and the fruit to leaf ratio per tree [20]. Furthermore, SSC values vary between different fruit and even for the same measurement position [12,21]. However, spectral scanning allows a large sample size to be obtained relatively fast. These measurements could provide a feasible alternative in the practice to labour intensive and costly laboratory analyses to gain a better idea of the distribution in SSC values.

Furthermore, standard ANOVA analyses are used at a particular moment in time (mostly at harvest) to determine the SSC distribution. This approach overlooks SSC developement over the course of time [22]. In the case of repeated measurements in agriculture and horticulture, mixed-effects models show clear advantages with respect to missing or unbalanced observations and different or restricted measurement periods [23]. When modelling is based on repeated measurements during fruit development, the longitudinal structure results in linear mixed-effect (LME) models to describe time-dependent changes linked to treatment effects and physiological influences. This class of LME models is a flexible subset of (generalized) regression models and can be used to model growth patterns in horticulture [24,25] and other research areas such as physical anthropology [26], clinical biometry [27] or ecology [28]. Modelling apple growth with expolinear, Gompertz and logistic [29,30] functions and adapted von Bertalanffy models [31] is common but SSC accumulation has been less frequently modelled. This is gradually changing through the use of in-depth biochemical analyses and the use of optical handheld sensors. Vis/NIR point spectrometers allow for repeated non-destructive spectral scanning on the same fruit. In a further classification process, longitudinal Vis/NIR data can enable modelling and classification of optimal harvest dates [32,33].

The present study focuses on non-destructive and longitudinal SSC accumulation in fruit in the orchard and the practical application of the above outlined methodology to set guidelines for their broader use. The carbohydrate physiology i.e., SSC accumulation during apple ripening was monitored within the experimental field treatments, reviewed from a user perspective and a statistical viewpoint. The results of the study were based on a large data set of Vis/NIR scans obtained over three study years.

This study investigates in detail (1) the number of calibration samples needed for a robust SSC prediction, (2) the effects of laboratory errors in wet chemistry analyses on PLSR model results, (3) the reliability of modelled SSC values in the orchard in comparison to standard laboratory tests of an independent sample and (4) time-dependent treatment effects on longitudinal SSC accumulation.

## 2. Results

The apple cropping seasons of 2016, 2017 and 2018 in Southwest Germany were distinctively different. A very wet spring with a light frost event during bloom was recorded in 2016. In 2017, severe frosts occurred during bloom in many European horticulture regions. At the Kompetenzzentrum Obstbau Bodensee, the number of trees available for research studies was reduced to those protected with heaters in plastic tents. Furthermore, 2018 was a relatively hot and dry year with 398 mm of precipitation between April to October compared to 956 mm and 730 mm for the same periods of 2016 and 2017, respectively. There were also clear differences between years in plant development for the growth stages (BBCH) flowering and fruit ripe for picking with differences of up to 12 days.

**Figure 1:**
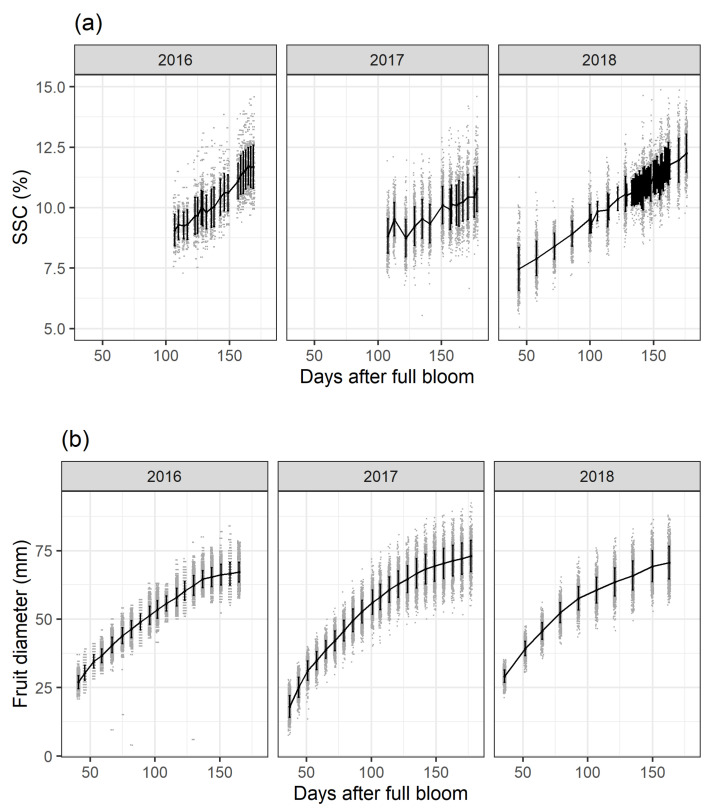
Soluble solids content (SSC) accumulation derived from the yearly calibrated (Section 2.1.1) partial least squares regression models (**a**) and fruit diameter growth (**b**) for the three study years and all treatments are shown. Mean values per measurement day are plotted as solid line, single values as grey dots and +/−standard deviations as black vertical bars.

SSC accumulation derived from the yearly calibrated PLSR model (Section 2.1.1) (Figure 1a) and fruit diameter growth (Figure 1b) are plotted as days after full bloom (DAFB) over the three study years. Fruit diameter and SSC were monitored at the same measurement intervals and for the same fruit. Fruit growth will not be further discussed here and serves only as additional background information on orchard data variance. SSC increases over time following a linear trend. In general, these data indicate a continuous mean SSC accumulation until apple maturity which is consistent with biochemical fruit analyses. In 2017 around 120 DAFB, the within-fruit variability during the time-series data acquisition shows either that the same fruit accumulate and degrade SSC between the scanning intervals or that the measurement environment negatively affected data acquisition. In 2016 and 2017, SSC had approximately the same values at around 120 DAFB. For 2018 the highest SSC values for the three study years were observed. In order to obtain a higher time-series resolution and larger time-series data for improved modelling in [33], data acquisition took place on a daily basis for 120-180 DAFB and SSC scanning started at 50 DAFB in 2018.

### 2.1. PLSR Calibration Models

#### 2.1.1. Multi-Year (2016–2018) Calibrated Model

**Figure 2:**
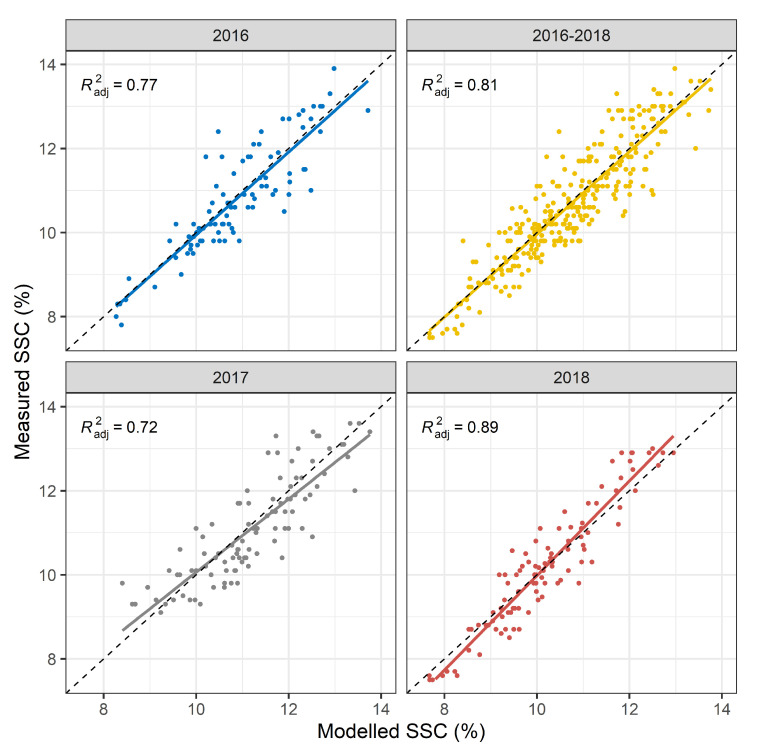
Regressions between laboratory measured and modelled % SSC. The calibration model was trained on 2016–2018 data. This model was thereafter evaluated with an independent validation data set for all years together and separately. Regression lines are plotted for each validation data set and adjusted prediction $R^{2}$ is displayed.Diagnostic plots

The multi-year model was built with calibration data from all study years (2016–2018). The multi-year model was subsequently validated with either a multi-year or the respective yearly data set.The multi-year model results in a root mean square error of prediction (RMSEP) of 0.65% SSC (adjusted $R^{2}$ of 0.77) in 2016, 0.67% SSC in 2017 (adjusted $R^{2}$ of 0.72), and 0.54% SSC in 2018 (adjusted $R^{2}$ of 0.89) compared to 0.62% SSC (adjusted $R^{2}$ of 0.81) over all three years combined (Figure 2). A look at the RMSEP and the adjusted prediction $R^{2}$ is usually not sufficient to determine the presence of systematic errors in the PLSR model. Fitting a linear regression to the prediction values resulted in a slight deviation from a diagonal line indicating the presence of small but negligible systematic errors (bias). More specifically, the multi-year PLSR model possibly underestimates or overestimates the frequency of particularly low or high SSC values in 2017 and 2018. Residual plots (data not shown) suggest a slightly heteroscedastic structure. In terms of model performance over all years, the multi-year PLSR model appears to yield reasonable predictions with minor restrictions.

#### 2.1.2. Year-Dependent Calibration Model Transfer to Other Years

Independent PLSR calibration models for each study year were calculated to check the transferability and adequacy of year-dependent PLSR model to other study years (500 observations in the calibration data set, 100 observations in the validation data set). Results suggest that yearly calibrated models perform best for scans taken within the same year (Table 1).

The multi-year PLSR model predictions (calibration data set) based on reference samples measured from all years in equal parts show a slight increase in the mean RMSEP values compared to the yearly calibrated prediction models. The calibration model based on 2016 data gives a RMSEP and standard deviation (sd) in bracket value of 0.61 (+/−0.05) % SSC for 2016 validation data compared to a RMSEP value of 0.68 (+/−0.05) % SSC for the multi-year model (2016–2018). In 2017 and 2018 similar results were obtained. Yet, all yearly calibrated models performed poorly in other years. Standard deviations were considerably higher when using validation data from years that were not part of the calibration data sets. This suggests two conclusions: first, yearly calibrated models tend to overfit the data and can hardly be used as general PLSR models. Second, as the range in SSC values in 2018 was wider than in 2016 or 2017, this increased range led to poor model performance for the 2016 and 2017 models which were not trained for particularly low or high SSC values.

### 2.2. Evaluation of the Training Data

#### 2.2.1. Effect of Sample Sizes

**Figure 3:**
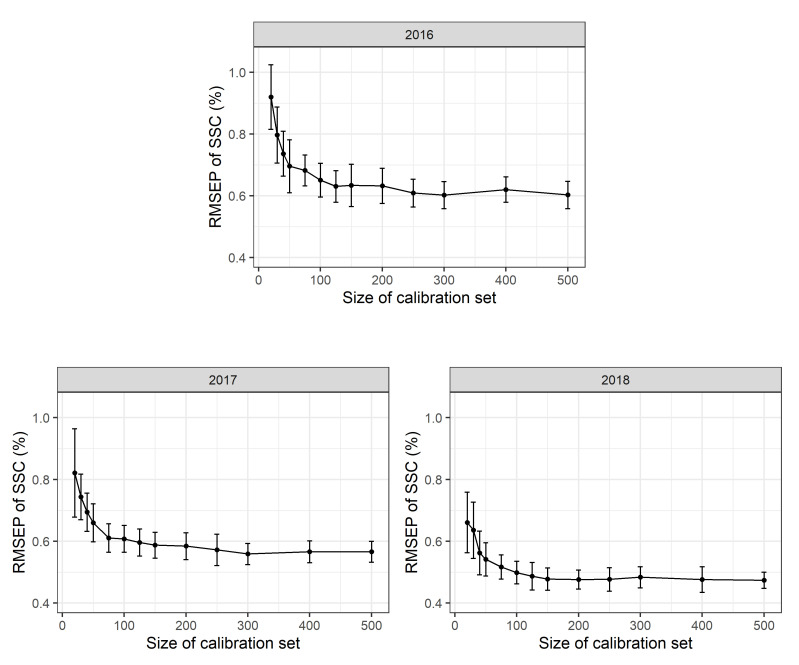
Root mean square error of prediction (RMSEP) in % soluble solids content (SSC) based on 2016, 2017 and 2018 partial least squares regression (PLSR) models to test the effect of reduced calibration sample sets. For each setting, 500 Monte Carlo simulation runs were performed. Mean and standard deviation for each point are shown and 500 observations in each calibration data set and 100 observations in each validation data set used.

To assess the effects of different calibration sample sizes, 500 repeated Monte Carlo simulation runs were performed. For each simulation run a stratified random sample with *n* = 20, 30, 40, 50, 75, 100, 125, 150, 175, 200, 300, 400 or 500 calibration measurements per year (corresponding to 60 to 1500 calibration measurements in total) was used as a calibration data set to calibrate a multi-year (2016–2018) PLSR model. The RMSEP was determined using a validation set with *n* = 200 observations for each year. The mean RMSEP of these Monte Carlo simulation runs and the standard deviations thereof are shown in Figure 3. PLSR models based only on 20 calibration scans per year show a mean RMSEP of 0.93 (+/−0.18 sd) % SSC in 2016, 0.91 (+/−0.17 sd) % SSC in 2017 and 0.95 (+/−0.28 sd) % SSC in 2018. PLSR models based on 100 calibration measurements per year result in a mean RMSEP of 0.70 (+/−0.06 sd) % SSC in 2016, 0.70 (+/−0.06 sd) % SSC in 2017 and 0.60 (+/−0.07 sd) % SSC in 2018. Based on 500 calibration measurements per year, a mean RMSEP of 0.67 (+/−0.05 sd) % SSC in 2016, 0.67 (+/−0.04 sd) % SSC in 2017 and 0.56 (+/−0.04 sd) % SSC in 2018 are obtained. As the 2018 calibration measurements include a higher proportion of scans taken during early fruit development, the larger range of SSC values available results in a supposedly lower RMSEP value. This fact, to a large extent, explains the apparent model improvement in 2018. Besides, the accuracy of laboratory calibration work may also have improved in the third year of the study. No differences in mean RMSEP values were detected for scans conducted at different temperatures in the laboratory with Monte Carlo simulation (∼10, 20 or 30 ${}^{\circ }$C).

#### 2.2.2. Effect of the Data Range

To assess the prediction quality at certain SSC values the RMSEP was also determined with 500 repeated Monte Carlo simulation (1200 observations in the calibration data, 300 observations in the validation data set). The multi-year model (2016–2018) was split into values of <9, 9–10, 10–11, 11–12, 12–13 and >13% SSC and resulted in a RMSEP of 0.59 (+/−0.08 sd), 0.55 (+/−0.05 sd), 0.57 (+/−0.04 sd), 0.65 (+/−0.06 sd), 0.73 (+/−0.08 sd) and 0.82 (+/−0.09 sd) % SSC, respectively. The analysis of the mean RMSEP shows signs of heteroscedasticity with a worse PLSR prediction for lower and especially higher SSC values. However, there was a smaller calibration data set at the beginning and end of fruit ripening.

In all years, mean RMSEP values were very high for a low number of calibration values and decrease rapidly up to *n* = 100 reference values per year with only a slight additional improvement in model adequacy as shown in the RMSEP for *n* > 100 calibration values per year. Standard deviations for the RMSEP values are rather high for small calibration sets and decrease with increasing sample size. This suggests that model accuracy might appear high in some cases “by chance”. This has two implications: first, the number of calibration measurements can be limited to a rather small number of observations per year and a reduced number of calibration measurements can be used in future experiments. Second, a certain prediction error seems to be inevitable with a given PLSR model no matter how many calibration measurements are available.

#### 2.2.3. Effect of Refractometer Errors

**Figure 4:**
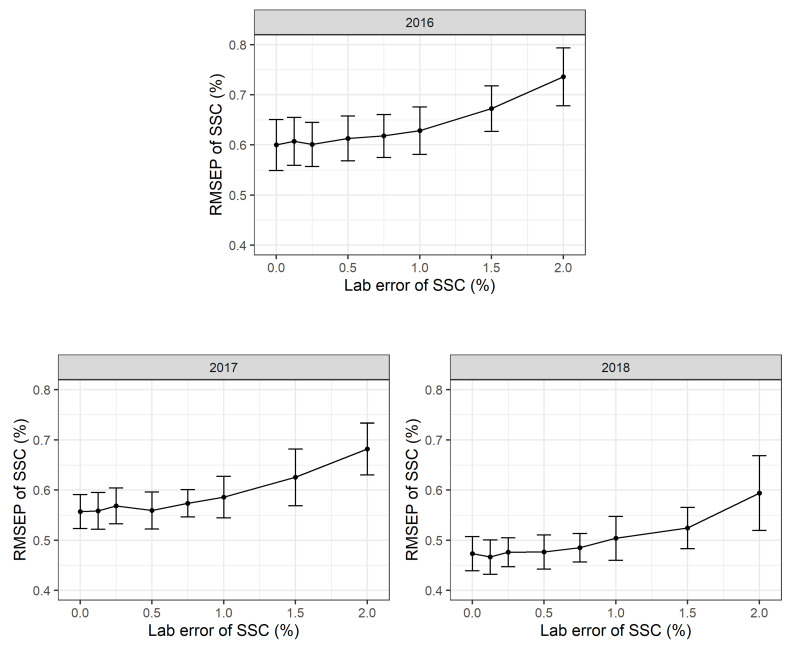
Root mean square error of prediction (RMSEP) in % soluble solids content (SSC) based on 2016, 2017 and 2018 partial least squares regression (PLSR) models to test for nonsystematic laboratory errors during wet chemistry analyses. For each setting, 500 Monte Carlo simulation runs were performed. Mean and standard deviation for each point are shown and 500 observations in each calibration data set and 100 observations in each validation data set used.

In this simulation procedure, additional normally distributed noise with a mean value of m = 0% SSC and standard deviations of s = 0, 0.1, 0.2, 0.3, 0.4, 0.5, 0.75, 1.0, 2.0% SSC was added to the laboratory reference values for each simulation run. The standard deviation of s = 0% SSC corresponds to the standard PLSR calibration model. All the simulation runs show highly robust PLSR models. Even moderate and substantial laboratory errors only increase the RMSEP values slightly (Figure 4). The RMSEP in 2016 increased from 0.67 (+/−0.05 sd) % SSC to 0.74 (+/−0.06 sd) % SSC with an additional laboratory error and a standard deviation of 2.0% SSC. The simulations show similar results for 2017 and 2018.

### 2.3. Use of LME Models to Describe SSC Accumulation

At any given time, the SSC values follow a linear trend with a normal distribution and a slight increase in variance during fruit development and maturation (Figure 1b). A breakdown by different orchard factors/experimental management treatments for tree sector, crop load, cell division temperature and calcium treatment for the 2018 season mainly suggests a clear effect of sector position, lower effects of crop load and temperature and no effect of calcium treatment (data not shown). These trends are consistent with the observations made for the 2016 and 2017 seasons. Three different models are considered to evaluate whether the specification of random effects and interaction terms yields a substantial improvement in model quality.

Model 1 is a fully-specified LME model with tree-specific and fruit-specific random intercepts, year, weeks after full bloom (WAFB), sector position, crop load, cell division temperature and calcium treatment as fixed effects and interactions between time and the above listed main effects. Model 1 is a fully-specified LME:

(1) $$y_{ijk}=\beta_{0}+u_{j,0}+u_{i,0}+\beta_{1}t_{k}+u_{j,1}t_{k}+u_{i,1}t_{k}+\beta_{X}X_{i}+\beta_{Y}X_{i}t_{k}+e_{ijk}$$

with a population intercept $\beta_{0}$, a population parameter $\beta_{1}$ for modelling a time-dependent linear accumulation trend, a time-independent population parameter $\beta_{X}$ for any other fixed effects such as treatment effects and sector position, a time-dependent parameter $\beta_{Y}$ for these fixed effects, a random tree-specific intercept $u_{j,0}$ with $u_{j,0}∼N\left(0,\sigma_{u_{j,0}}^{2}\right)$, a random tree-specific slope $u_{j,1}$ with $u_{j,1}∼N\left(0,\sigma_{u_{j,1}}^{2}\right)$, a random fruit-specific intercept $u_{i,0}∼N\left(0,\sigma_{u_{i,0}}^{2}\right)$, a random fruit-specific slope $u_{i,1}∼N\left(0,\sigma_{u_{i,1}}^{2}\right)$ and a random error term $e_{ijk}∼N\left(0,\sigma_{e}^{2}\right)$. Model 2 is nested in Model 1 without interaction terms ($\beta_{Y}=0)$:

(2) $$y_{ijk}=\beta_{0}+u_{j,0}+u_{i,0}+\beta_{1}t_{k}+u_{j,1}t_{k}+u_{i,1}t_{k}+\beta_{X}X_{i}+e_{ijk}$$

Model 3 corresponds to a fully-specified linear regression model without random effects (with $u_{j,0}=u_{i,0}=u_{j,1}=u_{i,1}=0$) and serves as a baseline model to compare the effects of the LME modelling. Model 3 is a standard linear regression model without random effects:

(3) $$y_{ijk}=\beta_{0}+\beta_{1}t_{k}+\beta_{X}X_{i}+\beta_{Y}X_{i}t_{k}+e_{ijk}$$

The fixed time and treatment effects for any given fruit *i* from tree *j* were calculated as:

(4) $$E\left(y_{ijk}|i,j\right)=\beta_{0}+\beta_{1}t_{k}+\beta_{X}X_{i}+\beta_{Y}X_{i}t_{k}$$

Using Akaike information criterion (AIC) and Bayesian information criterion (BIC) as indicators of goodness of fit, both LME models (Models 1–2) are clearly favourable over the standard linear regression (Model 3). Based on 17,004 observations, Model 1, 2 and 3 result in an AIC of 27,896, 29,154 and 35,708 and a BIC of 28,074, 29,255 and 35,871, respectively. The root mean square error (RMSE) was 0.48, 0.50 and 0.69% SSC for Model 1, 2 and 3, respectively. Further information about regression coefficients and time-dependent treatment effect of Model 1 can be obtained from Table 2 and Table 3. Of these two mixed-effects models, Model 1 seems to give the best fit which suggests that the specification of interaction terms is appropriate to reflect the spatial and temporal dependencies between observations. Model 1 shows highly significant effects for year, WAFB, sector crop load and cell division temperature and their interactions. No significant effect is observed for the calcium treatments.

The time-dependent effects of different treatment levels are displayed in Table 2. Fruit from the light crop load treatment show increasing SSC values throughout fruit development. At the end of cell division (∼40 DAFB), only small differences between different crop loads (range of 0.09% SSC between light and high crop load) and cell division temperature regimes (0.16% SSC between cold and warm temperatures) can be observed. Close to harvest (140 DAFB), relatively large differences in SSC can be seen between different levels of tree sector (range of 0.89% SSC between bottom and top sector) compared to only minor differences in SSC for different crop loads (range of 0.42% SSC between light and high crop load) and only negligible effects for different temperature regimes (range of 0.12% SSC).

### 2.4. Sensitivity Analysis of the Experimental Setup

Data collection in large orchard trial designs is labour and cost intensive. Therefore, it is of interest to investigate whether reduced sample sizes lead to different results. SSC values were derived from the multi-year PLSR calibration model based on laboratory reference measurements. The influence of reduced sample sizes and unbiased laboratory measurement errors of 1.0% SSC on research results was investigated with LME Model 1. Different simulation settings are presented in Table 3.

Three settings are discussed and the standard Model respectively altered: Model A shows the effects of a reduced number of trees (100 trees within three years, same number of fruit per tree). Model B is based on a reduced number of fruit (500 fruit within three years, same number of trees). While the first setting with less experimental trees makes if possible to have additional experiments in the same orchard block, the second setting reduces the number of working hours per tree. Additionally, Model C shows the effect of an increased but unbiased measurement error (additional white noise of 1.0% SSC) in the SSC measurements. As expected, Models A–C show increased standard deviations of all estimates compared to the multi-year SSC model due to a reduced sample size (for Models A and B) or an increased measurement error (Model C). In Model C, the cell division temperature and calcium treatment are not marked as “statistically significant” due to increased measurement errors. In most cases the effect of sample size remains comparable to the multi-year SSC model.

In summary, these simulations show the possibility of reduced sample sizes when the focus is on treatments with large effects. In order to detect small differences between different treatments, large sample sizes are still required, especially in the presence of measurement errors due to increased *t*-values of the estimates.

### 2.5. A Practical Comparison of Spectral and Conventional Laboratory Methods to Determine SSC at Harvest

Traditional destructive laboratory samples for SSC were taken at harvest from eight apples per tree sector, treatment (2 or 3× levels) and repetition (3×). At the same time, the last non-destructive scans in the orchard were taken from an independent batch of approx. seven apples, scanned and postprocessed with the yearly calibrated PLSR model. Mean destructive laboratory values for 2016, 2017, 2018 and all study years from 2016 to 2018 were 11.3, 11.1, 12.3 and 11.7% SSC, respectively (2016: +/−0.52 sd, *n* = 63; 2017: +/−0.55 sd, *n* = 9; 2018: +/−0.53 sd, *n* = 50). The non-destructive samples were 11.8, 10.7, 12.0 and also 11.7% SSC, respectively (2016: +/−0.85 sd, *n* = 63; 2017: +/−0.70 sd, *n* = 27; 2018: +/−0.56 sd, *n* = 68). There is a higher variance for the PLSR modelled SSC values as compared to the laboratory values. The mean difference of each treatment level for the two methods is 0.5% SSC for all study years. The obtained values from the two approaches were not biased.

## 3. Discussion

Varying weather conditions during the three study years resulted in different SSC values at harvest which is in accordance with the literature [34,35,36]. Using time-series data in the orchard offers the possibility to see a linear carbohydrate development in the form of SSC accumulation over time. For 2017, the severe frost year, the SSC increase showed a larger variance (Figure 1a), as was also seen for fruit growth (Figure 1b). Non-destructive technologies can provide researchers with new tools to study fruit physiology or offer the possibility to use these values in digital orchard management information systems to predict and manage fruit quality, as seen for fruit diameter [37]. The effects of field treatments and physiological differences were directly related to the developmental stage of the fruit. Differences in tree sector position and crop load [1,38] caused increasingly large differences in SSC during fruit development and negligible differences due to early season temperature (Δ 2 ${}^{\circ }$C to ambient). Differences in sector position influence SSC early in the season whereas crop load effects increase steadily during fruit development.

Up until now the practical application of non-destructive scanning in apple research experiments has been restricted due to the intensive amount of laboratory work necessary to obtain reference samples and to the unknown precision of PLSR calibration models in the orchard. The results of the PLSR calibration only partially depend on the number and precision of the reference laboratory measurements. The results suggest that special emphasis should be placed on scanning fruit at low and high SSC values at the beginning and end of each season to cover a wider range of possible SSC values within a particular growing season. These results have some practical implications and suggest that even a considerably reduced sample size (100 samples) leads to comparable results, although the standard deviations of the estimates increase with reduced sample size. It suggests that repeated laboratory reference measurements of the same fruits to increase the accuracy of reference values lead to almost negligible improvements of the PLSR calibration models. These simulation results are consistent with standard results from statistical measurement error theory for response variables [39]. Moreover, in future experimental designs a reduced number of field scans would be sufficient to detect SSC differences between the treatments. A classical experimental field design with blocks and repetitions did not play a role in the LME modelling, which relaxes some limitations of the classical variance analysis framework and provides a more flexible way to adapt to temporal, spatial and tree-specific dependencies. A precision horticulture approach beyond research trials to monitor fruit SSC development on large sample numbers aligned to orchard structure should be possible.

The accuracy and robustness of the PLSR models was examined in great detail and only showed minor limitations to their broader use for our purposes. Yearly calibrated models cannot be generalised to other years, but multi-year models can be used for the same orchard and cultivar as was also seen in Peirs et al. (2003) [21]. The practical comparison between all laboratory based destructive measurements and the non-destructive orchard SSC data collection showed that the independent apple selection was unbiased and for the apple cultivar ‘Braeburn’ there was no difference between the two methods for determining SSC values at harvest in the orchard. In the future, however, new developments with model transfer methodology [40] together with neural networks or other ‘big data’ applications may facilitate the wider use of non-destructive sensor based SSC predictions for apples.

Our results may not be generalised to other apple cultivars or fruit species and to other sites or other climate regimes. However, since all effects are comparatively large and consistent with a literature review, additional measurements would probably confirm the overall effects. Analyses of dry matter content which can also be obtained by PLSR models were not considered in this study. As the number of non-destructive sensors available for horticultural practice and research is expected to increase in the coming years, longitudinal data will be available in ever greater quantities. The collaboration of horticultural science, computer science and statistics will avoid the collection of data as an end in itself and allow for new insights into currently hidden patterns of fruit physiology and development.

## 4. Materials and Methods

### 4.1. Experimental Setup

This research took place at the Kompetenzzentrum Obstbau-Bodensee (47${}^{\circ }$46′01.9″ N 9${}^{\circ }$33′23.3″ E) in the Lake Constance region of Southwest Germany using the apple cultivar ‘Braeburn’ *Malus domestica*. A randomised field design with treatments of crop load (light, standard, heavy), calcium spraying (with, without) and cell division temperature (ambient, Δ + 2 and Δ− 2 ${}^{\circ }$C) were used. Each tree was divided into three sectors of ∼1.25 m height each for the bottom, middle and top. Apple phenological growth stages were recorded following the BBCH code scheme [41]. The experimental design (treatments and scanning number/frequency) varied during the different study years. For a detailed description of the field experiments see [33,42].

### 4.2. SSC Sampling

Around June drop, one representative fruit per tree and sector was selected, marked and repeatedly measured (scanned) until harvest. Fruit were scanned on the equatorial and sun side with a handheld portable Vis/NIR device (F-750, Felix Instruments, Camas, WA, USA). The device had a 3 nm spectral sampling over a 310–1100 nm spectral window, a spectral resolution of 8–13 nm and corrected each scan for background daylight. The spectral range of 729–975 nm was used in the PLSR models to predict SSC. Fruit were replaced by a similar nearby fruit, if the fruit was lost or was not representative.

Orchard sampling was performed weekly in 2016 between 15 August and 16 October for *n* = 198 fruit from *n* = 33 trees. A total of *n* = 3994 scans were performed. In 2017, sampling took place weekly between 3 August and 30 October for *n* = 603 fruit from *n* = 96 trees. A total of *n* = 5957 scans were made. In 2018, *n* = 473 fruit from *n* = 146 trees were measured biweekly between 6 June and 25 October. In total, *n* = 7087 scans were recorded. In 2018, data acquisition took place on a daily basis for 120–180 DAFB and SSC scanning started at 50 DAFB.

### 4.3. PLSR Models

Reference measurements combined both destructive wet chemistry results and non-destructive spectral scans. A sample of *n* = 30 reference fruit were taken regularly over the fruit development and maturation periods from nearby trees in the same block and around the same field at scanning time to ensure the transferability of the calibration model to the SSC of sampled fruit. In total, *n* = 599 fruit in 2016, *n* = 211 fruit in 2017 and *n* = 333 fruit in 2018 were selected. Non-destructive spectral reference measurements were performed at different temperatures (∼10, 20, 30 ${}^{\circ }$C) to help adjust for temperature induced changes in hydrogen bonding [43]. The number of reference measurements is given as a total of *n* = 1639 observations in 2016 (529 observations at 10 ${}^{\circ }$C, *n* = 583 observations at 20 ${}^{\circ }$C, *n* = 527 observations at 30 ${}^{\circ }$C), in 2017 *n* = 631 observations (210 observations at 10 ${}^{\circ }$C, *n* = 211 observations at 20 ${}^{\circ }$C, *n* = 210 observations at 30 ${}^{\circ }$C) and in 2018 *n* = 984 observations (*n* = 328 observations at each temperature level). Destructive wet chemistry SSC measurements were obtained with a refractometer (Atago, Tokyo, Japan). PLSR models were postprocessed on a year- and site-specific basis.

The original models were built with the Felix model builder software (v1.3.0.177). Additional PLSR models were fitted using the R package pls [44]. Spectral data was transformed using second derivative spectra from 729 nm to 975 nm. The maximum number of principle components was set to 7 and the models were validated with leave-one-out cross validation methods. The reference data set was split into a calibration data set to train the PLSR model and a validation data set which was only used to test the prediction quality. A stratified random sample was drawn for each year to generate equal parts for all years. In total, *n* = 1200 observations were used as calibration data and *n* = 300 for validation data, if not stated otherwise. The RMSEP and adjusted prediction $R^{2}$ were used to describe the model performance and goodness of prediction. Reference measurements were taken as the longitudinal observations took place in the orchard. Therefore, we assume that the RMSEP for the validation data corresponds to the RMSEP of the SSC sampled fruit which could not be chemically analysed destructively due to the longitudinal structure of the study.

### 4.4. Monte Carlo Simulations

Monte Carlo simulations were used to assess PLSR model sensitivity to changes in input parameters and effects of sample size [45]. Measurement accuracy using standard laboratory analyses was simulated with repeated random samples. For simulations a modified and randomly sampled calibration set was generated without replacement. Reference measurements were split into a calibration data set with 1200 observations and a validation data set with 300 observations. The modified calibration sets were used in an automatic Monte Carlo simulation process to calculate the RMSEP and adjusted $R^{2}$ values for each setting. Each setting was repeated *n* = 100 times to calculate mean RMSEP values and standard deviations.

For sample size analyses, calibration sets with a reduced sample size were sampled for each year and all years combined. For laboratory errors, calibration sets were sampled for each year and all years combined. Laboratory errors were assumed to be unbiased and normally distributed. Additional normally distributed error terms (white noise) with different magnitudes were added afterwards.

### 4.5. Longitudinal LME Models

LME models (hierarchical regression models, nested linear models, multi-level regression models) are a subset of generalized regression methods to analyse repeated time-correlated and cluster-correlated observations [46]. DAFB and WAFB were used for time-dependent analyses in each year.

All time-correlated observations on a single fruit were part of a natural cluster of observations which shared the same fruit-specific and tree-specific characteristics. A hierarchical (nested) data structure was therefore applied. The LME model combined population-specific and subject-specific (spatial variation in the orchard) random effects.

The specification of the random effects needs special consideration as the longitudinal data structure makes two adaptions necessary: first, a random effect is given by a fruit-specific dependency as these observations are correlated over time (fruit-specific intercept and slope). Second, a random effect is necessary due to tree-specific dependency for all fruit from the same tree. Therefore, with respect to the natural dependency of observations from the same tree, a tree-specific intercept and slope were specified. Two random effects were modelled in addition to fixed effects which affect all fruit simultaneously. Details are specified in the previous sections. Modelling was done with the R package lme4 which provided various functions for fitting, analysing and evaluating mixed-effects models in a linear, generalised linear and nonlinear framework [47,48]. The restricted maximum likelihood method and full maximum likelihood method were used to estimate parameters. The R package lmerTest was used to approximate the degrees of freedom and calculate *p*-values for mixed-effects models using Satterthwaite’s method [49]. Yet no emphasis is placed on the interpretation of these *p*-values, as there is an unresolved statistical discussion about their theoretical applicability [50]. Coefficients of fixed effects with a *t*-value (ratio of estimate and its standard deviation) of less than −2 or greater than 2 were considered statistically significant. Model choice was based on the AIC and the BIC both of which use the log-likelihood ratio and describe model quality by adjusting the goodness of fit with a penalization term for model complexity [51,52]. RMSEP was used to compare model predictions and observations.

### 4.6. Mann–Whitney–Wilcoxon Test

A Mann–Whitney–Wilcoxon test was conducted in R to compare the modelled SSC values from the field scans based on the PLSR modelsto destructively measured fruit in the laboratory. Refractometer values showed a normal distribution, whereas PLSR modelled SSC were not normally distributed and Mann–Whitney–Wilcoxon test was used. For the laboratory samples the top half of a fruit batch of eight apples was mixed in the laboratory with a conventional fruit blender.

## 5. Conclusions

In summary, the non-destructive temporal development of SSC accumulation could contribute new insights into apple fruit carbohydrate physiology. The present study linked an in-depth statistical analysis of large data sets with horticultural knowledge in order to test the application of ‘Braeburn’ SSC prediction models with a special focus on model transferability and accuracy.

- In terms of model performance over all years, the multi-year PLSR model appeared to be reasonable with minor restrictions for especially low and high SSC predictions. However, independent yearly calibration models performed best for the same year.
- A sample size of *n* = 100 fruit for a yearly PLSR model with a wide range of SSC values seems to be sufficient.
- Differences in sector position and crop load resulted in increasingly large differences in SSC during fruit development and offer the possibility for further physiological studies.

## Figures and Tables

**Table 1:** Root mean square error of prediction (RMSEP) of % soluble solids content (SSC) to test the transferability of calibration models after 500 Monte Carlo simulation runs per point (500 observations in each calibration data set, 100 observations in each validation data set. Mean and standard deviation (in brackets) for each point are shown.

Training Data Set	Validation Data Set of Respective Years			
	2016–2018	2016	2017	2018
2016–2018	0.65 (0.05)	0.68 (0.05)	0.68 (0.04)	0.57 (0.04)
2016	0.90 (0.16)	0.61 (0.05)	1.00 (0.30)	1.02 (0.21)
2017	0.83 (0.12)	0.95 (0.23)	0.57 (0.04)	0.90 (0.14)
2018	0.95 (0.10)	1.19 (0.20)	1.01 (0.08)	0.48 (0.03)

**Table 2:** Time-dependent treatment effects in 2017 on % soluble solids content (SSC) accumulation at different stages of fruit development given in days after full bloom (DAFB). The baseline configuration (Base) corresponds to fruit from the bottom sector of a tree with medium crop load without alterations of cell division temperature. Displayed effects are bottom, middle, top sectors and light, standard (stand.), heavy crop load and cold, ambient (amb.), warm cell division temperature treatments. SSC values of the respective effects need to be added or subtracted to the base value.

DAFB	Base in % SSC	Sector		Crop Load		Temperature	
		Middle	Top	Light	Heavy	Cold	Warm
40	5.54	0.21	0.53	0.02	−0.07	0.08	0.16
60	6.43	0.25	0.60	0.06	−0.09	0.05	0.13
80	7.31	0.30	0.67	0.11	−0.11	0.01	0.11
100	8.19	0.34	0.75	0.16	−0.13	−0.02	0.08
120	9.08	0.39	0.82	0.20	−0.15	−0.06	0.06
140	9.96	0.43	0.89	0.25	−0.17	−0.09	0.03

**Table 3:** SSC model simulations with a reduction of sample size and different measurement errors. Multi-year model (MYM) corresponds to a linear mixed-effect model with all data from 2016–2018 included (LME Model 1), Model A was reduced to samples from 100 trees of the MYM, Model B was reduced to 500 fruit samples of the MYM and Model C had an unbiased error of 1.0% SSC added to the MYM. The estimate % SSC (standard deviation) is stated with a significance code with *** <0.001, ** <0.01, * <0.05. The number of observations, different fruit and trees is shown.

	Multi-Year Model	Model A	Model B	Model C
Observations (N)	17,004	7457	6777	17,004
Fruit (N)	1274	540	500	1274
Trees (N)	237	100	211	237
Intercept	3.78 *** (0.084)	3.72 *** (0.115)	3.90 *** (0.127)	3.86 *** (0.157)
Year 2017	1.47 *** (0.096)	1.58 *** (0.136)	1.45 *** (0.148)	1.51 *** (0.183)
Year 2018	1.85 *** (0.081)	2.04 *** (0.114)	1.87 *** (0.125)	1.85 *** (0.150)
Week	0.31 *** (0.003)	0.31 *** (0.005)	0.31 *** (0.005)	0.31 *** (0.007)
Middle sector	0.11 * (0.054)	0.00 (0.078)	−0.03 (0.087)	0.01 (0.100)
Top sector	0.39 *** (0.054)	0.38 *** (0.078)	0.35 *** (0.089)	0.423 *** (0.101)
Cold temperature	0.15 (0.089)	−0.02 (0.126)	−0.03 (0.131)	−0.05 (0.152)
Warm temperature	0.21 ** (0.065)	0.15 (0.090)	0.26 ** (0.095)	0.09 (0.107)
Light crop load	−0.07 (0.064)	−0.05 (0.096)	−0.17 (0.094)	−0.11 (0.110)
Heavy crop load	−0.03 (0.069)	−0.11 (0.094)	−0.18 (0.101)	−0.01 (0.117)
Without calcium	0.17 (0.140)	0.00 (0.175)	0.43 (0.230)	0.03 (0.227)

## Data Availability

All data reported here is available from the authors upon request.

## Abbreviations

The following abbreviations are used in this manuscript:

AIC	Akaike information criterion
ANOVA	Analysis of variance
BIC	Bayesian information criterion
DAFB	Days after full bloom
LME model	Linear mixed-effects model
NIR	Near-infrared
PLSR	Partial least squares regression
RMSEP	Root-mean-square error of prediction
sd	Standard deviation
SSC	Soluble solids content
Vis	Visible
WAFB	Weeks after full bloom
